# Obstructing Stage IV Adenocarcinoma of the Transverse Colon in a Young Patient With Vitiligo

**DOI:** 10.7759/cureus.42679

**Published:** 2023-07-30

**Authors:** Ellie Mueller, Zeba Shaik, David Addepalli, Sara Malik, Patrick Schiefelbein

**Affiliations:** 1 Medical School, New York Institute of Technology College of Osteopathic Medicine, Jonesboro, USA; 2 General Surgery, Northwest Health, Siloam Springs, USA

**Keywords:** adenocarcinoma of the transverse colon, early onset cancer, colon cancer prevention, adenocarcinoma, laparoscopic colon resection, auto-immunity, vitiligo, colorectal cancer

## Abstract

Advanced colorectal cancer, while uncommon, can occur in a young patient. We present a rare case of advanced transverse colon cancer in a young patient with vague symptoms and unique comorbid conditions, while reviewing the literature on colorectal cancer and its association with autoimmune conditions. With a recent increase in the incidence of colon cancer in young patients, further research is needed as to whether colorectal cancer screening is warranted in younger cohorts outside of current recommendations and guidelines. Investigations are needed into the factors that may explain this and the public health interventions that can be employed to improve colon cancer prevention. The objective of this report is to highlight the importance of recognizing alarming symptoms and raise awareness of the increasing incidence of early-onset colon cancer in young patients.

## Introduction

Colorectal cancer remains the third-most common neoplastic malignancy and second-most fatal for both male and female sexes in the United States as of 2022 [1]. With concerted efforts to increase screening rates, the incidence among older adults has steadily declined since the 1980s. The opposite trend has occurred among individuals under the age of 50, where incidence has risen 1.2% to 3.0% annually since the mid-1980s [2]. Following a rigorous review, advisory organizations such as the American Cancer Society, American College of Gastroenterology, and the United States Preventative Service Task Force (USPSTF) have amended their guidelines, encouraging surveillance to begin at age 45 rather than 50 for patients at average risk [3-5]. 

Early detection of colon cancer carries significant prognostic value. Broadly speaking, colon cancer survival rates are predicted by staging at the time of diagnosis. According to the Surveillance, Epidemiology, and End Results (SEER) database [1], localized colon cancers carry a five-year survival of 91%, or 73% for regional tumors. The five-year survival drops to 13% for distant metastases. The combined rate is 63%. Timely diagnosis is aided by obtaining screenings at the suggested regular intervals. Young patients at average risk typically depend on their clinical symptoms to prompt diagnostic workup. Therefore, it is not surprising that rates of localized and metastatic diagnoses are higher in younger patients, compared to older patients [6]. 

Advanced colon cancer may present with non-specific gastrointestinal symptoms, creating diagnostic ambiguity. The rarity of colon cancer compared to other benign gastrointestinal disorders may delay diagnosis, as other causes may be explored preferentially. Additionally, the location of the disease can affect the diagnostic timeline and prognosis. Distal sigmoid lesions have a better prognosis because they are detected early in the course due to more obvious clinical symptoms. However, proximal lesions, including the 10% located in the transverse colon, are most likely to have high tumor grade and lymph involvement [7]. 

Additionally, vitiligo associations with internal dysregulations, malignancies, and other autoimmune conditions have been discussed in the literature for almost a century. Most recently, literature has demonstrated trends suggesting that vitiligo may play a protective role against general cancer incidence [8]. However, this trend has been debated by several population studies with contradictory results [8-11]. 

In this case report, we will discuss the aforementioned literature trends and relate them to our unique case presentation of advanced adenocarcinoma in a young male with vitiligo. We will discuss the challenges posed when approaching young patients with vague gastrointestinal symptoms.

This article was previously presented as a poster presentation at the 2023 American College of Osteopathic Surgeons - Medical Student Section meeting on April 29, 2023.

## Case presentation

Initial presentation

**Figure 1:**
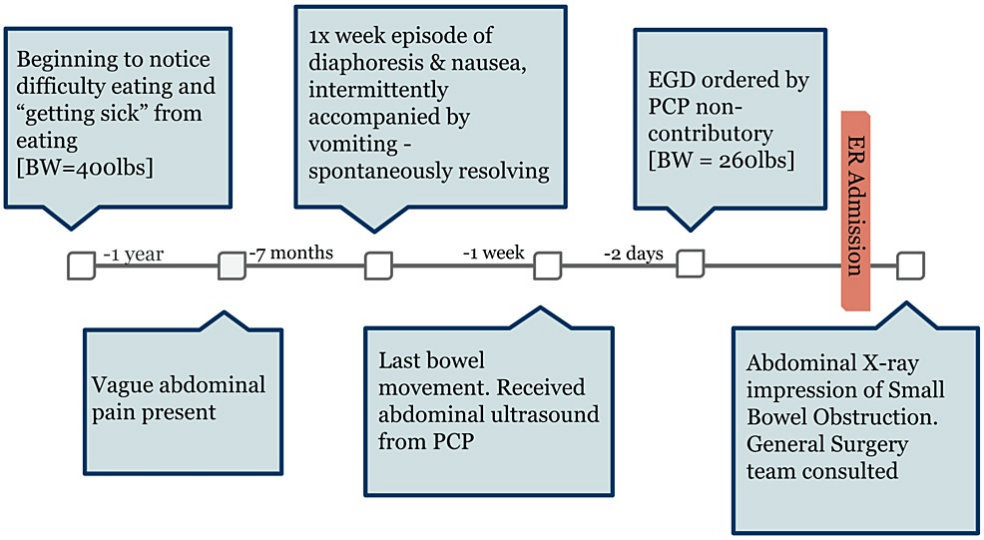
Timeline of patient’s symptoms and initial workup leading to hospital admission BW: body weight; PCP: primary care physician; EGD: esophagogastroduodenoscopy

A 32-year-old male with visible vitiligo presented to the emergency department with chief complaints of abdominal pain, nausea and vomiting, and an inability to pass flatus or stool for one week. Prior to arriving at the emergency department, the patient had undergone an esophagogastroduodenoscopy (EGD) and an abdominal ultrasound, which were both non-contributory. Additionally, the patient reported experiencing vague abdominal pain and spontaneous self-resolving periods of extreme nausea and diaphoresis, described as "dizzy spells", for the last seven months. These episodes were accompanied by periods of constipation without melena or hematochezia. Further questioning revealed a 140-pound weight loss, from an initial 400 pounds, in one year along with occasional "getting sick" from eating. Figure 1 demonstrates the consolidated timeline.

The patient denied any family history of colon cancer, as well as any other cancers. The patient had a history of type 2 diabetes mellitus and hypertension, well controlled with metformin and lisinopril, respectively. He denied a history of Crohn’s disease or ulcerative colitis. He admitted to smoking one half-pack of cigarettes per day since his early 20s, and noted skin changes due to vitiligo began around age 19. There were no family members with known vitiligo or autoimmune conditions. 

A physical examination of the abdomen in the emergency department revealed diffusely minimal tenderness to palpation with mild abdominal distention. The remainder of the physical exam was unremarkable. Laboratory test values in the emergency department were within normal limits. 

Investigations

**Figure 2:**
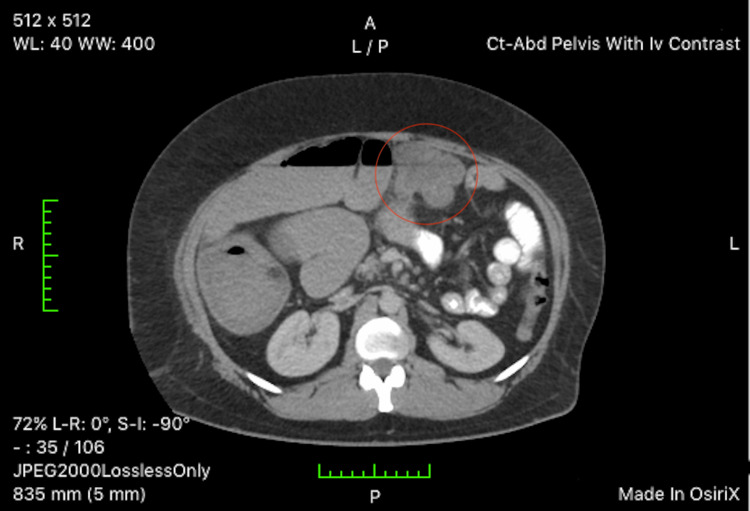
Axial view of obstructing, lobulated colonic mass (red circle). Note air-fluid levels proximal to mass.

**Figure 3:**
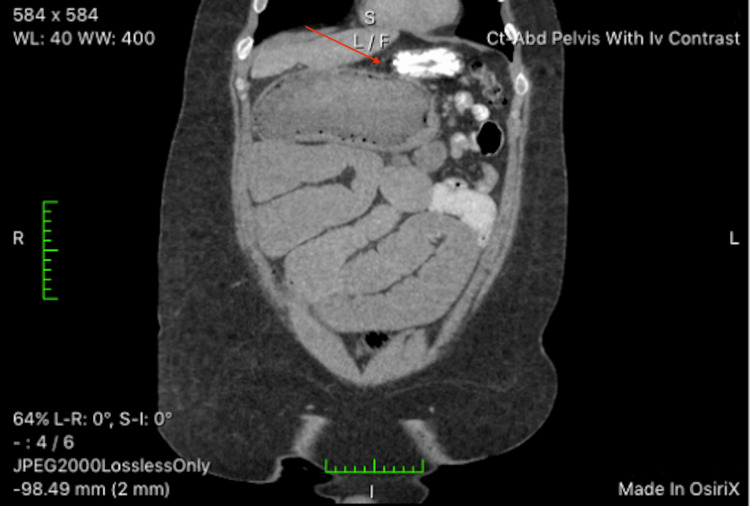
Coronal view of dilated loops of colon and small bowel proximal to lesion, with compressed bowel distal. Note clear mid-transverse colonic transition zone delineated by red arrow.

Upon presentation to the emergency department, an erect X-ray was obtained. The X-ray demonstrated generalized abdominal distention with possible small bowel obstruction, prompting surgical consultation. Preoperative carcinoembryonic antigen (CEA) levels were obtained by the surgical team and were elevated at 3.1 ng/mL (reference range 0-2.9 ng/mL). Subsequently, a nasogastric tube was placed with prompt bilious output. Intravenous fluid resuscitation was started, along with morphine and ondansetron as needed. Computed tomography (CT) of the abdomen and pelvis was obtained with contrast dye enhancement. CT results (Figures 2, 3) demonstrated a lobulated, obstructing mass in the middle transverse colon, multiple enlarged pericolonic lymph nodes, and one peritoneal nodule in the upper anterior abdomen. There was significant distension of the proximal middle colon and middle to distal small bowel, air-fluid levels, and compression of colonic segments distal to the mass. Surgical hemicolectomy was scheduled for the following day for the removal of the insulting lesion.

**Figure 4:**
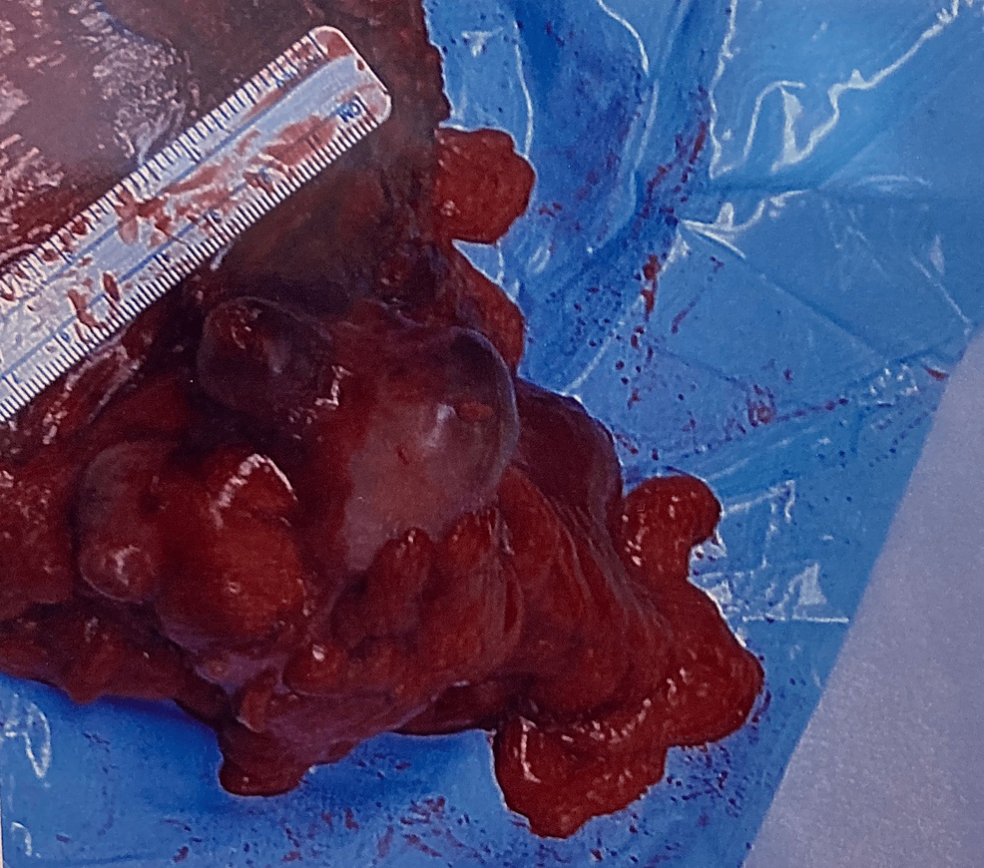
Image of intra-operative specimen on the transverse colon measuring 75 x 45 x 15 mm.

The patient underwent an extended right hemicolectomy with an omental biopsy for suspected metastasis. The colonic specimen and omentum were sent for histopathological processing. The tumor itself was transmural with extensive mesenteric involvement. The resected colonic specimen had negative margins with a focally close implant to the visceral peritoneal surface. The pathology report revealed a 75 x 45 x 15 mm lobulated mass that was moderate to poorly differentiated with mucinous features (Figure 4). Lymphovascular invasion was extensive, with two of 31 lymph nodes identified. Mesenteric deposits were present and the omentum was distantly involved. The pathology report confirmed a pT3 Nib M1b stage IVB adenocarcinoma. Genetic testing of the resected mass confirmed a low/stable microsatellite instability as well as retained MSH6 and other mismatch-repair genes.

Outcome and follow-up

**Figure 5:**
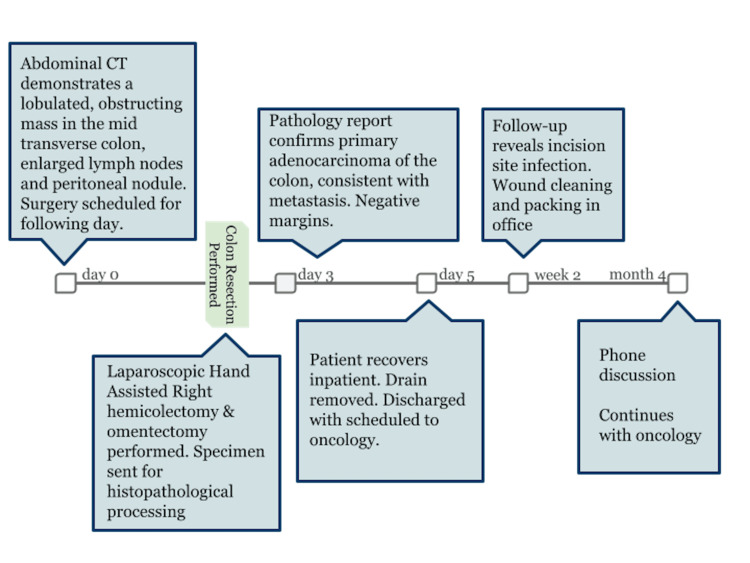
Timeline of diagnostic workup upon hospital admission, surgical management, and recovery

The patient recovered in five days and had no postoperative complications prior to hospital discharge. Figure 5 demonstrates the timeline.

The patient returned to the clinic for a follow-up three weeks after the surgery with a cutaneous surgical site infection. The site was packed with gauze soaked in Dakin’s solution and redressed. The patient was instructed to pack and dress the wound daily. Metronidazole and levofloxacin were prescribed. He returned two weeks later with a well-healing wound.

A month after the surgical treatment, the patient had an oncology consultation. A week after oncology consultation, a port was placed for a systemic chemotherapy regimen consisting of leucovorin calcium, fluorouracil, and oxaliplatin (FOLFOX). The follow-up four-month whole-body positron emission tomography (PET)/CT imaging was negative for metastatic disease. The patient’s younger siblings and children were advised to obtain a surveillance colonoscopy regardless of genetic testing. Initially, he tolerated chemotherapy but later acquired new-onset peripheral neuropathy. Additionally, he was treated for anxiety with fluoxetine and lorazepam. New-onset microcytic anemia was treated with iron supplementation. 

After discharge, the patient was able to return to activities of daily living. However, he admitted to new-onset depression during his time of surgery and recovery. With the support of his caregivers, family members, and a colorectal cancer awareness support group, he felt able to battle this depression and take on a positive outlook on his life. 

## Discussion

Colorectal cancer in young patients is more likely to be aggressive and late-staged, making early detection imperative to increase survival rates [12]. Currently, the United States Preventive Services Task Force (USPSTF) states that colon cancer screening starts at 45 years of age in those without risk factors or family history of grade B colon cancer, and 10 years before the time of colon cancer diagnosis in direct family members [5]. 

Colon cancers are theorized to develop from polyps undergoing dysplastic changes over a 10-15 year period before developing into malignancy. Franks and Slansky suggest vitiligo in the setting of known diagnosed melanoma may act as a defense against malignancy via self-targeting melanocytes [13]. While the immune system is capable of generating “anti-cancer” responses, it is also capable of promoting oncogenesis. Chronic immune activation can cause tissue damage and can become malignant. The review by Franks and Slansky importantly notes that those with a primary malignancy may develop autoimmune or autoimmune-like diseases. In this case, our patient’s cancer could have occurred prior to age 19 at the onset of depigmentation, raising the question of whether the vitiligo began in response to oncogenesis. While unlikely, it cannot be ruled out. 

Efforts to understand the rising incidence of colon cancer in young patients are ongoing. Research suggests the molecular basis of early-onset tumors may differ from late-onset tumors [14]. While familial and genetic factors may contribute to early-onset cases, sporadic occurrences are more predominant. Additionally, certain lifestyle risk factors such as alcohol consumption, smoking, physical inactivity, and excess body weight have also been linked to cancer. The impact of alcohol and smoking is less likely, as consumption has declined in young age cohorts over the same period of rising incidence. However, trends in obesity rates coincide with the rise of cancer cases for the age groups of concern, primarily adults under the age of 45 [15]. Dietary patterns including high consumption of processed meats and low fiber, along with a sedentary lifestyle are independent contributors to both obesity and colon cancer. Studies suggesting the role of “Western” diets of high-fat, low-fiber foods as contributors to colonic inflammation have been corroborated by studies involving immigrants [2]. Generational “jumps” in colon cancer rates were observed in a study of Japanese migrants following their immigration and adoption of the United States lifestyle [16]. 

A report from 2008 discusses a patient with vitiligo who was diagnosed with four synchronous colorectal cancers at 17 years of age, and systemic lupus erythematosus at 16 years of age [17]. This particular case involved a bi-allelic mutation of MSH6 as well as high microsatellite instability, pointing towards a Lynch-like syndrome. However, the pathology report on our patient’s biopsy confirmed a low/stable microsatellite instability as well as retained MSH6 and other mismatch-repair genes. A genetic root cause is less likely, and a sporadic development of colon adenocarcinoma in this young patient is more probable. 

Recent studies suggest that vitiligo plays a protective role against cancer incidence, particularly skin cancer. Nationwide studies in Taiwan and Korea have contradictory theories of how vitiligo interacts with malignancy [8,11]. First, the study by a Taiwan lab group concluded that there are increased risks of certain cancers in patients with vitiligo [11]. The study showed 44.3% of cancers diagnosed in persons with established vitiligo were less than 40 years old. Colorectal cancers had a higher incidence than expected, with a greater increase in women.

A research team in Korea demonstrated a reduced risk of internal malignancies in those with vitiligo compared to their control group [8]. This team also found that young patients 20-39 years old had a reduced risk of colorectal cancer than those aged greater than 60 years. 

A third, independent research team analyzed these trends using a Mendelian randomization with strict protocol for controls. They concluded vitiligo was causally associated with reduced risks of many cancers; however, this trend was not seen in colon or rectal cancers [10]. According to their findings, they proposed that the autoimmune effects of vitiligo may not be cell-specific, but rather may have diverse impacts on different organ systems.

A more recent nationwide study in Taiwan corroborated the significantly reduced risk of overall internal malignancies in patients with vitiligo across all ages and gender, but most significantly in males [9]. This team acknowledges several factors that may influence the data, including that more hospital visits for someone with vitiligo may lead to a greater chance of cancer detection, leading to the overestimation of cancer rates in those with vitiligo. However, there was a lower incidence found in the study, which suggests a possible underestimation of the negative association. A pitfall of the surveillance studies is the inability to take into account lifestyle and environmental risk factors.

Despite the evidence from large, high-power studies demonstrating a lower incidence of colorectal cancer among those with diagnosed vitiligo, the physiological mechanism linking the two conditions is unknown. Understanding the pathophysiological connection could reveal new and potential therapeutic mechanisms.

Some studies suggest that vitiligo serves as a protective factor against cancer incidence and markedly decreased incidences of internal malignancies [8]. Yet, as demonstrated by our report, this trend is not a rule. While there may be a reduced risk, no protective immunomodulatory factors against internal malignancies have yet been proven. Further surveillance studies must be conducted in various populations to further explore this trend. Patients with symptoms, regardless of age, should be screened for colon cancer, as early detection can be diagnostic and curative.

While colon cancer affects less than 1% of young adults, the rise in incidence is a cause for concern. The lack of clarity around the factors driving these changes suggests the trends will continue. By highlighting the case presented here, we hope to amplify the need for increased clinical awareness of colon cancer cases in young patients. Further research is needed to determine the need for expanding the screening protocols among patients at average risk. Due to the increased incidence of colorectal cancer, accessibility to noninvasive imaging will be necessary to discover malignancy early. We have analyzed screening guidelines and suggested solutions to posed difficulties in detecting colon cancer in the young population. These include future studies targeting younger cohorts to investigate the cost-benefit analysis of early colorectal cancer screening, as well as validation of low-cost mass screening modalities such as fecal immunochemical tests (FIT) or multi-target DNA tests.

## Conclusions

Advanced colorectal cancer in young patients is uncommon, but can still occur. Increasing incidence of colon cancer in young patients warrants further research to investigate factors that may explain this phenomenon. Autoimmune conditions such as vitiligo may not have a lower correlation or causative effect on internal malignancies and additional research is needed in order to establish a clear significance of vitiligo and lower risk of internal malignancies. Given the abundance of technologies available for screening, increasing public health education initiatives and awareness, in both physicians and patients, could increase early detection and treatment of gastrointestinal malignancy.
